# Nonsurgical Treatment of Two Periapical Lesions with Calcium Hydroxide Using Two Different Vehicles

**DOI:** 10.1155/2014/901497

**Published:** 2014-07-15

**Authors:** Seema Dixit, Ashutosh Dixit, Pravin Kumar

**Affiliations:** ^1^Department of Conservative Dentistry & Endodontics, Seema Dental College, Rishikesh 201410, India; ^2^Department of Periodontics, Seema Dental College, Rishikesh 201410, India

## Abstract

Calcium hydroxide is used extensively as an intracanal medicament in endodontics for many years. It is used in various clinical situations such as to promote apexification, to repair perforation, to enhance healing of periapical lesions, to control root resorption, and to control exudation in teeth with persistent periapical inflammation. This paper presents a case report in which Ca(OH)_2_ was used as an intracanal medicament for treatment of periradicular lesions using two different vehicles in two different teeth of same patient.

## 1. Introduction 

The main goal of endodontic therapy is to eliminate or at least achieve a significant reduction of microorganisms present in the root canal system. It is well recognized that chemomechanical instrumentation alone is unable to completely disinfect the root canal system [1]. The bacteria surviving root canal instrumentation proliferate between appointments. Therefore, the use of intracanal medication with antimicrobial activity between appointments has been recommended to eliminate possible persistent microorganisms, particularly in case of pulp necrosis with periradicular bone loss [2]. Calcium hydroxide with its antimicrobial property has been widely used in endodontics for interappointment intracanal dressing. It has been demonstrated that treatment with calcium hydroxide as an interim dressing in the presence of large and chronic periapical lesions can create an environment more favorable to healing and encourage osseous repair [3].

## 2. Mechanism of Antimicrobial Activity of Calcium Hydroxide

Most of the root canal microbes are unable to survive in the highly alkaline environment provided by calcium hydroxide. Several bacterial species commonly found in infected root canals are eliminated after a short period when in direct contact with this substance [4].

Antimicrobial activity of calcium hydroxide is related to release of hydroxyl ions in an aqueous environment. Hydroxyl ions are highly oxidant-free radicals that show extreme reactivity resulting in the damage to bacterial cytoplasmic membrane, protein denaturation, and damage to bacterial DNA [1, 4–7].

It has been asserted that all biological actions of calcium hydroxide progress by the ionic dissociation in calcium ion and hydroxyl ion (Estrela 1995) [8]. The vehicle plays the most important role in overall process because it determines the velocity of ionic dissociation causing the paste to be solubilized and resorbed at various rates by the periapical tissues and from within the root canal (Fava 1991) [9].

In general three types of vehicles are used: aqueous, viscous, or oily. The first group is represented by water soluble substances including water, saline, dental anesthetics, Ringer's solution, aqueous suspension of methyl cellulose or carboxymethyl cellulose, and anionic detergent solution. When calcium hydroxide is mixed with one of these substances, Ca^2+^ and OH^−^ ions are rapidly released [10]. This type of vehicle promotes a high degree of solubility. The root canal may become empty in a short period, delaying the healing process. From a clinical standpoint, this means that root canal must be redressed several times until the desired effect is achieved, thereby increasing the number of appointments.

Some viscous vehicles are water soluble substances that release Ca^++^ and OH^−^ ions more slowly for extended periods. They promote lower solubility of the paste when compared with aqueous vehicles, for example, glycerine, poly ethylene glycol, and propylene glycol [9].

Oily vehicle are nonwater soluble substances that promote the lowest solubility and diffusion of paste within the tissues. Paste containing this type of vehicle may remain within the root canal for longer than the paste containing aqueous or viscous vehicle, for example, olive oil, silicone oil, camphor, metacresylacetate, and some fatty acids [9].

This paper presents a case of the treatment of two contralateral teeth of the same patient with calcium hydroxide using two different vehicles.

## 3. Case Report

**Figure 1:**
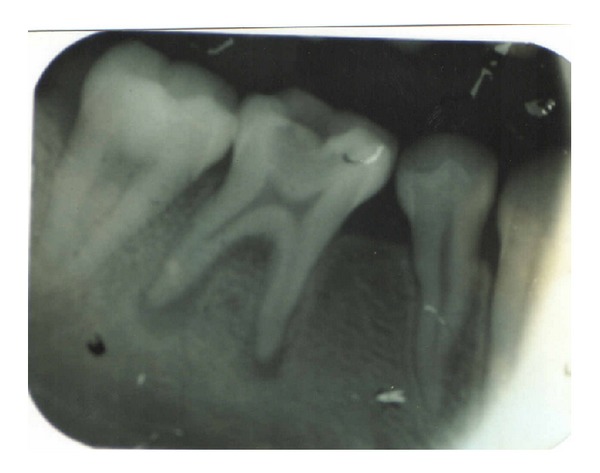
Before operation (36).

**Figure 2:**
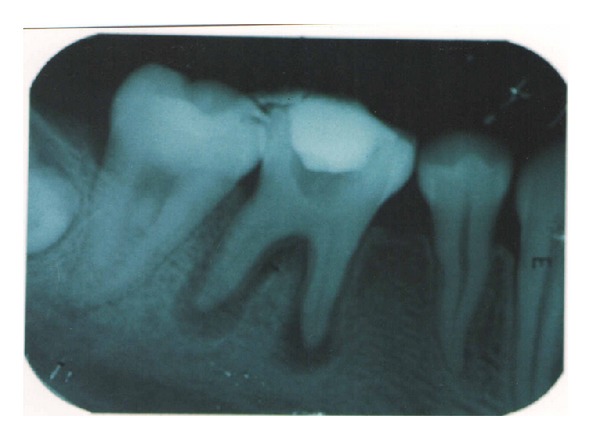
Ca(OH)_2_ paste placed (36).

**Figure 3:**
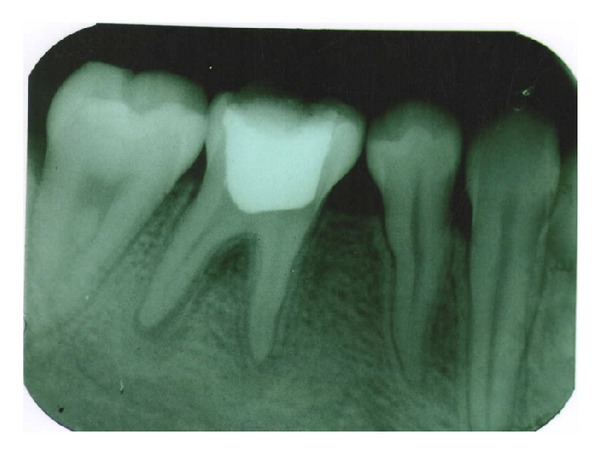
Healing at 9 months (36).

**Figure 4:**
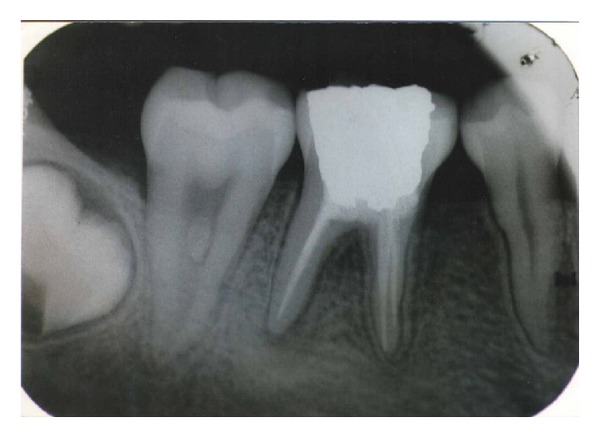
Obturation (36).

**Figure 5:**
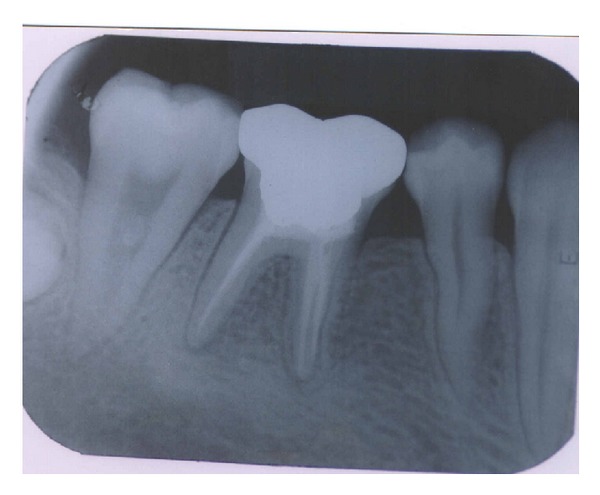
Crown placed (36).

**Figure 6:**
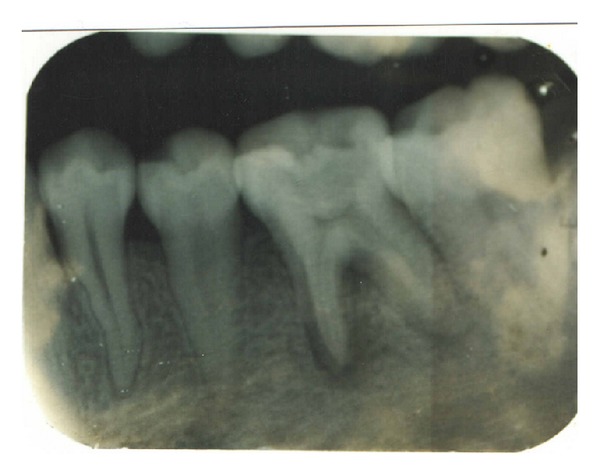
Before operation (46).

**Figure 7:**
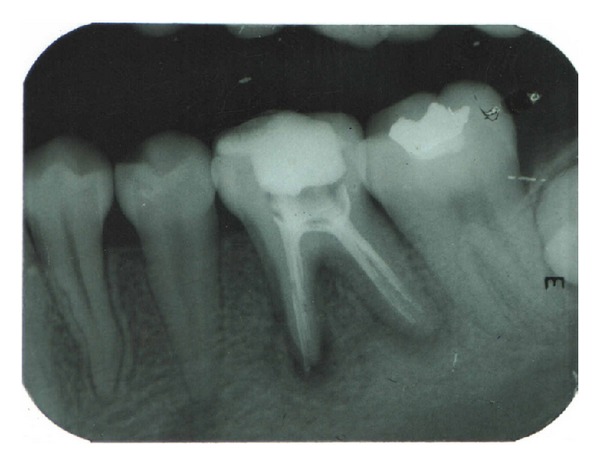
Metapex placed (46).

**Figure 8:**
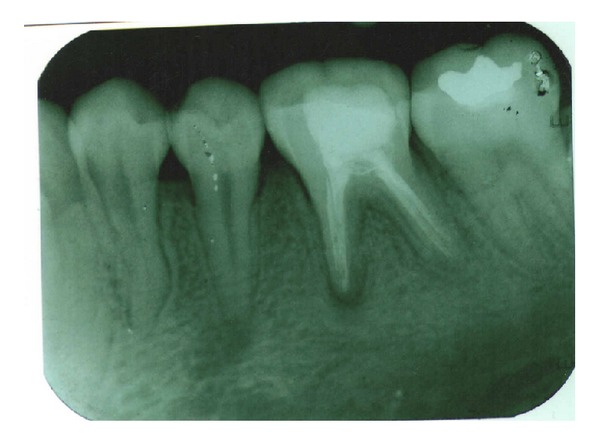
Radiograph showing healing after 9 months (46).

**Figure 9:**
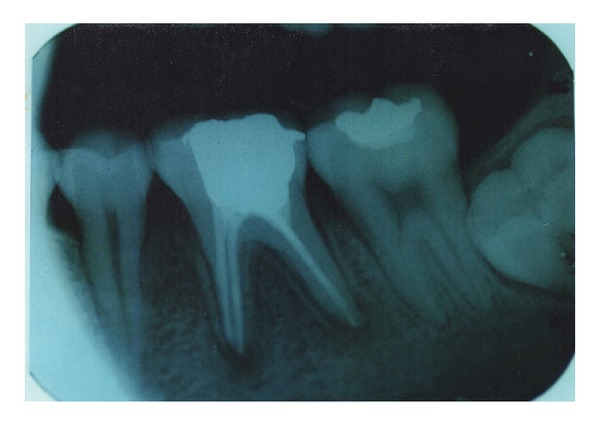
Obturation (46).

**Figure 10:**
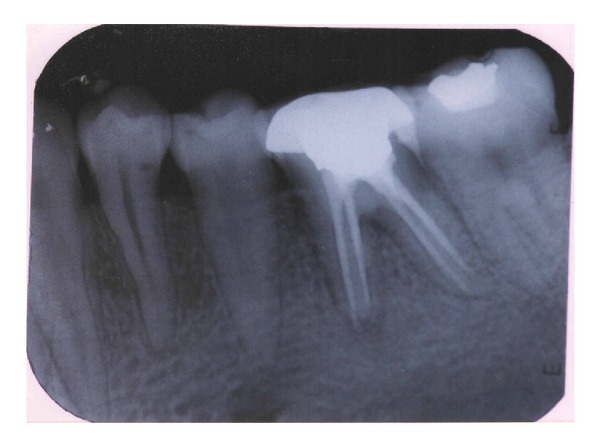
With crown (46).

A 17-year-old patient came to the Department of Conservative Dentistry and Endodontics, ITS Dental College Muradnagar, Ghaziabad, with complaint of pain in both sides in relation to the posterior teeth of lower jaw. On oral examination, it was found that 36 and 46 were cariously exposed. On radiographic examination, a large radiolucency in enamel and dentin approaching pulp was seen. The periapical area showed radiolucency in the mesial and distal roots of 36 and 46 (Figures 1 and 6). Root canal treatment was decided in both teeth. Access opening was done in both teeth. It was at this time that the decision was taken to compare the response of calcium hydroxide with two different vehicles, that is, saline and silicone oil, in this patient. Following the working length estimation in 36, a thorough chemomechanical preparation was performed. The root canals were irrigated with combination of sodium hypochlorite and sterile saline solution. The canals were dried with sterile paper points. Calcium hydroxide powder was taken on glass slab, mixed with saline solution, and slurry was made. With the help of appropriate size of lentulospiral, calcium hydroxide was placed in each canal until the paste was seen at the canal orifice and after that pulp chamber was packed with calcium hydroxide paste. The access cavity was sealed with intermediary restorative material and a radiograph was taken (Figure 2). The same procedure was followed in 46 till the drying of canal. After the canals were dried with paper points, metapex syringe was inserted in mesiobuccal canal and the material was injected into the canal. The metapex was further applied using appropriate size lentulospiral until the canal was completely filled with metapex. The procedure was repeated with the other two canals. The completed root canal filling with metapex was verified with the help of radiograph (Figure 7). The access cavity was sealed with intermediary restorative material. Radiographs were taken at 3-month interval. The calcium hydroxide paste in 36 was refreshed monthly till the radiographic evidence of healing of periapical lesion. The metapex in 46 was changed after every three months. Both teeth showed satisfactory radiographic healing after nine months (Figures 3 and 8). After nine months, removal of Ca(OH)_2_ paste and metapex from 36 and 46 was done using copious amount of sodium hypochlorite and saline and final rinse with EDTA. Obturation was done in both teeth (Figures 4 and 9) followed by postendorestoration and crown preparation and placement (Figures 5 and 10).

## 4. Discussion

Microorganisms are the cause of apical periodontitis and their elimination from the root canal space during root canal treatment results in predictable healing of apical pathosis. Unfortunately, the complete elimination of bacteria by instrumentation alone is unlikely to occur. In addition, pulp tissue remnants may prevent microorganisms from being entombed as well as have a negative impact on the root filling in terms of its physical properties and adaptation to the canal walls. Thus, some form of irrigation and disinfection is necessary to kill and remove microorganisms, their by-products, and residual tissue as well as remove the smear layer and other debris from the canal system. Such chemical (therapeutic) treatments of the root canal can be arbitrarily divided into irrigants, canal rinses, and interappointment medicaments. Calcium hydroxide is included in the intracanal medicaments group.

Since its introduction to dentistry calcium hydroxide (Hermann 1920) has been indicated to promote healing in many clinical situations [11, 12]. Although the overall mechanism of action of calcium hydroxide is not fully understood, its antimicrobial activity is influenced by its speed of dissociation into calcium and hydroxyl ions in a high pH environment which inhibits enzymatic activities that are essential to microbial life, that is, metabolism, growth, and cellular division. Ca(OH)_2_ is often used to effect periapical healing by combination of its antimicrobial activity and its ability to promote hard tissue formation and periodontal healing [1, 4–7].

When calcium hydroxide is mixed with suitable vehicle, a paste is formed for use in endodontics.

The type of vehicle has a direct relationship with the concentration and the velocity of ionic liberation as well as with the antibacterial action when the pasted is carried into the contaminated area [10, 13].

Different vehicles have been added to calcium hydroxide in an attempt to enhance to its antimicrobial activity, biocompatibility, ionic dissociation, and diffusion.

In the present paper, two vehicles were employed one saline and other silicone oil.

When Ca(OH)_2_ is mixed with saline solution, Ca^++^ and OH^−^ are rapidly released when the paste comes in direct contact with the tissue and tissue fluids; it is rapidly solubilized and resorbed by macrophages; the root canal becomes empty in a short period. Some studies consider 4-week period to be a reasonable time interval to expect effective therapeutic benefits from Ca(OH)_2_ saline dressings [2, 14–16]. So, the dressing in 36 was replaced monthly till periapical healing was evident radiographically.

The other Ca(OH)_2_ paste used was metapex containing Ca(OH)_2_, silicone oil, and iodoform. Oily vehicle promotes the lowest solubility and diffusion of the paste within the tissues. So paste may remain within the root canal for longer. Thus, in 46 the paste was changed once in three months till the periapical healing was evident [9, 17].

Radiographs were taken three monthly. Considerable healing was observed after 9 months in both cases (Figures 3 and 8). After 9 months, both teeth, that is, 36 and 46, were obturated followed by crown preparation and cementation.

The periapical lesions in both teeth were successfully treated nonsurgically with no variation in healing duration and pattern except number of redressings were more in 36 using saline as vehicle.

## 5. Conclusion

Calcium hydroxide has a great value in endodontics, being indicated for several clinical conditions. In the present case, two different formulations of calcium hydroxide were used to treat periapical lesions. Despite the vehicle used, calcium hydroxide proved to be an effective intracanal medicament and resulted in successful resolution of the periapical lesions.
